# Beyond Traditional Approaches: A Pilot Study Exploring the Role of Injection Mitomycin C on the Surgical Resection Bed in Oral Cancer Treatment

**DOI:** 10.7759/cureus.42200

**Published:** 2023-07-20

**Authors:** Sandeep Ghosh, Sanjay M Desai, Bonny Joseph, Vinod Dhakad, Amar Jain, Elroy Saldanha, Dhruv Patel, Soumya Singh, Saurav Ghosh, Anjali Yadav

**Affiliations:** 1 Surgical Oncology, Sri Aurobindo Medical College & PG Institute, Indore, IND; 2 Anaesthesiology, Sri Aurobindo Medical College & PG Institute, Indore, IND; 3 Anaesthesiology, Employees' State Insurance Corporation Hospital, New Delhi, IND; 4 General Surgery, Index Medical College, Hospital & Research Center, Indore, IND

**Keywords:** locoregional recurrence, head and neck oncology, pilot project, mitomycin c (mmc), extranodal involvement, oral cancers

## Abstract

Background

Oral cavity cancer ranks sixth among all cancers worldwide. India has the most oral cancer cases and accounts for one-third of the global oral cancer burden. Oral cavity cancer is known to be associated with an elevated likelihood of locoregional recurrences, which account for the bulk of post-surgery and radiotherapy treatment failures. Mitomycin C (MMC) is an antineoplastic and antibiotic agent that is administered topically rather than intravenously to treat bladder and intraperitoneal tumors to avoid recurrences. This study aimed to investigate the use of injection MMC as a local application on surgical resection beds for patients undergoing surgery for oral cancer and to assess its efficacy in preventing regional recurrences.

Methodology

In this prospective, interventional, pilot study, patients were assigned randomly to two groups using simple randomization. Group A involved the application of two gauze pieces soaked with MMC injection. Group B involved the application of two gauze pieces soaked with a 10% betadine solution. During the pectoralis major myocutaneous flap harvest procedure for reconstruction, two gauze pieces soaked with either injection MMC solution (20 mg MMC in 20 mL of 0.9% normal saline) or 10% betadine solution were placed on the surgical resection bed for a 45-minute contact period. Patients were evaluated daily in the postoperative period for local complications. Regular follow-up visits were scheduled for 15 months of follow-up.

Results

After exclusions at various phases, the final analysis included 50 patients in Group A and 50 patients in Group B. Minor complications, specifically blackening of the skin flap in the neck resulting in surgical site infections, were observed in 16% (eight patients) of the MMC group and in 6% (three patients) of the betadine group (p = 0.1997) patients. In the MMC group, two (4%) patients experienced locoregional recurrences at three months, four (8%) patients at six months, six (12%) patients at nine months, eight (16%) patients at 12 months, and 10 (20%) patients at 15 months of follow-up. In contrast, locoregional recurrences occurred in two (4%) patients in the betadine group at three months, six (12%) patients at six months, nine (18%) patients at nine months, 12 (24%) patients at 12 months, and 15 (30%) patients at 15 months. Although the difference in locoregional recurrences between the two groups was not statistically significant, there was a trend of decreasing locoregional recurrences in the MMC group relative to the betadine group as the duration of follow-up increased. In the subgroup analysis of patients with pathological extranodal extension (ENE), only 10 of 18 patients with ENE in Group A (55.55%) experienced a recurrence, whereas all 12 patients with ENE in Group B (100%) experienced a recurrence within the same time frame. This difference in locoregional recurrence rates between the two groups was statistically significant, with a p-value of 0.0100.

Conclusions

Our study demonstrated that the local administration of MMC on surgical resection beds may lower the risk of locoregional recurrences in patients with oral cancer, especially those with ENE. These findings contribute to the ongoing efforts to enhance treatment strategies and patient outcomes for this challenging malignancy.

## Introduction

Oral cavity cancer ranks sixth among all cancers worldwide. India has the most oral cancer cases and accounts for one-third of the global oral cancer burden. Oral cancer poses a grave health problem for nations going through economic transition [1]. Oral cancer is a significant health concern in India, as it is one of the most prevalent forms of cancer affecting a sizable population. Due to their extensive exposure to risk factors, the low-income population is at the greatest risk. Tobacco use, particularly in the form of smokeless tobacco, is the most common cause of oral cancer, particularly in developing nations like India. Other than tobacco, the consumption of paan-containing leaves of piper betel, areca nut, lime, catechu, and cinnamon is the primary cause of oral cancer [2]. Kerala has the lowest incidence of oral cancer in India, while West Bengal has the highest incidence [3].

The standard treatment for oral cavity cancer consists of surgery, radiotherapy, and chemotherapy. The combination of surgery, chemotherapy, and radiation therapy can increase overall survival, especially for patients with advanced oral malignancies. Nevertheless, roughly one-third of patients who undergo surgery and adjuvant therapy are likely to experience a local or regional recurrence [4]. Oral cavity cancer is known to be associated with an elevated likelihood of locoregional recurrences, which account for the bulk of post-surgery and radiotherapy treatment failures. The aggressive and invasive behavior of recurrent oral cancer poses a formidable clinical challenge. Chemotherapy is the only treatment option for recurrent oral cancer when neither salvage surgery nor re-irradiation is possible, but its efficacy is limited due to the development of drug resistance.

Mitomycin C (MMC) is an antineoplastic and antibiotic agent isolated from the *Streptomyces caespitosus* soil bacterium. It is administered intravenously for the treatment of upper gastrointestinal, anal, breast, and bladder malignancies. MMC has additionally been applied topically as opposed to intravenously for the treatment of bladder and intraperitoneal tumors. As a large molecule that is not swiftly absorbed systemically and is rapidly eliminated by the kidneys, it has a favorable toxicity profile and a low systemic absorption rate. It has a 2 mm local penetration and can be administered topically to the intraperitoneal cavity and bladder [5]. Using all of this information, we decided to use injection MMC as a local application on surgical resection beds for patients undergoing surgery for oral cancer and to study its effectiveness in preventing locoregional recurrences.

## Materials and methods

This prospective, interventional, pilot study was conducted between January 2020 and August 2021 at the Department of Surgical Oncology, Sri Aurobindo Medical College, and PG Institute, Indore, India. Before participation, each patient provided written informed consent. The study was conducted in accordance with the ethical guidelines outlined in the 2013 revision of the 1964 Declaration of Helsinki and was approved by the institutional ethics committee (approval number: SAIMS/IEC/08/20).

The primary objective of this study was to evaluate the feasibility of using MMC-soaked gauze as a local application to reduce the risk of locoregional recurrences in surgically treated oral cancer patients. The secondary objective was to evaluate and document the local side effects associated with the use of MMC-soaked gauze in this patient population.

This study included individuals who were over 18 years old and were diagnosed with oral cavity squamous cell carcinoma, confirmed through histological examination. They were undergoing composite resection with the need for pectoralis major myocutaneous (PMMC) flap reconstruction. To be eligible for the study, patients needed to have an Eastern Cooperative Oncology Group (ECOG) performance status of 0, 1, or 2.

The exclusion criteria, on the other hand, included individuals who had previously received neoadjuvant chemotherapy, patients with recurrent oral cancer, previously operated cases, those with an ECOG score of 3 or higher, and those with thrombocytopenia, coagulation disorders, or bleeding disorders.

All patients underwent comprehensive evaluations, including routine blood tests and contrast-enhanced computed tomography of the neck. Individual tumors were staged using the eighth edition of the American Joint Committee on Cancer staging manual. After completing all necessary preoperative evaluations, surgery was scheduled for the patients.

Patients were assigned randomly to two groups using simple randomization determined by the toss of a fair coin. Group A involved the application of two gauze pieces soaked with MMC injection. In Group B, two gauze pieces soaked with a 10% betadine solution were applied.

**Figure 1:**
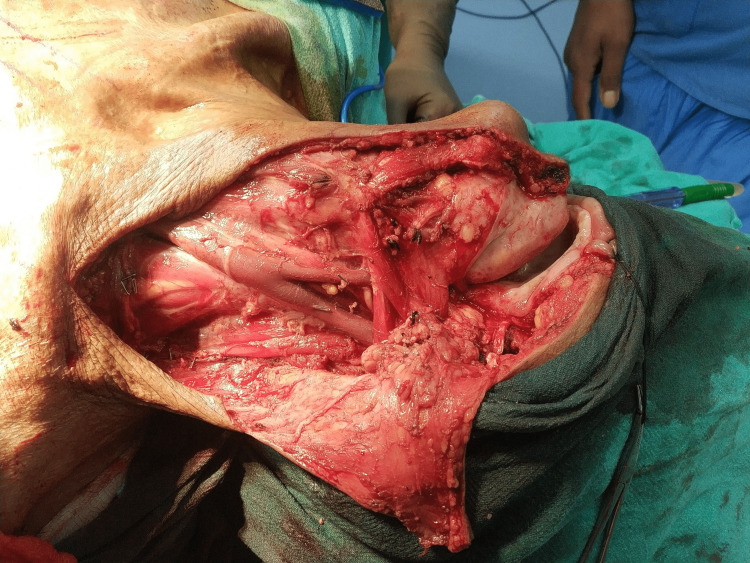
Surgical resection bed after oral cavity cancer composite resection.

**Figure 2:**
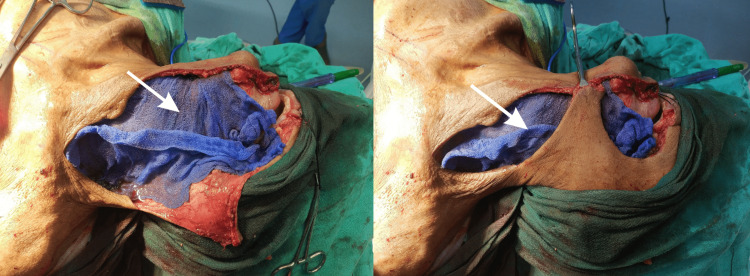
Local administration of mitomycin C-impregnated gauze (white arrow) on the surgical resection bed.

**Figure 3:**
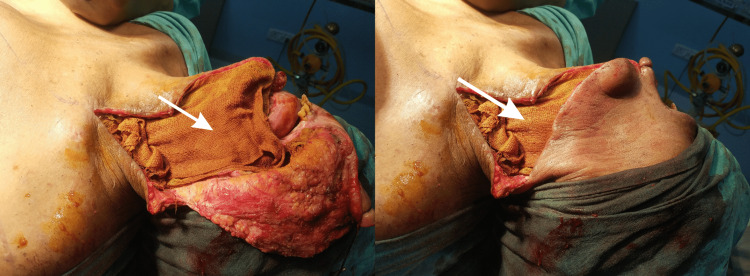
Local administration of betadine-impregnated gauze (white arrow) on the surgical resection bed.

During the PMMC flap harvest procedure for reconstruction, two gauze pieces soaked with either injection MMC solution (20 mg MMC in 20 mL of 0.9% normal saline) or 10% betadine solution were placed on the surgical resection bed for a 45-minute contact period (Figures 1-3).

Patients were evaluated daily during the postoperative period for local complications such as flap necrosis, infection rates, and wound dehiscence throughout the treatment course. Following surgery, recommendations for adjuvant treatment were made based on the final histopathology report and National Comprehensive Cancer Network (NCCN) guidelines.

In accordance with the institutional protocols, regular follow-up visits were scheduled monthly for the first year and then every two to three months for the second year for a total of 15 months of follow-up. Events such as locoregional tumor recurrence were documented for both groups during this period.

Statistical analysis

The statistical analysis of the data was performed using the INSTAT software (GraphPad Prism software, La Jolla, CA, USA). The chi-square test was employed to determine the relationship between variables. A p-value of less than 0.05 indicated a statistically significant correlation between the variables under investigation.

## Results

**Figure 4:**
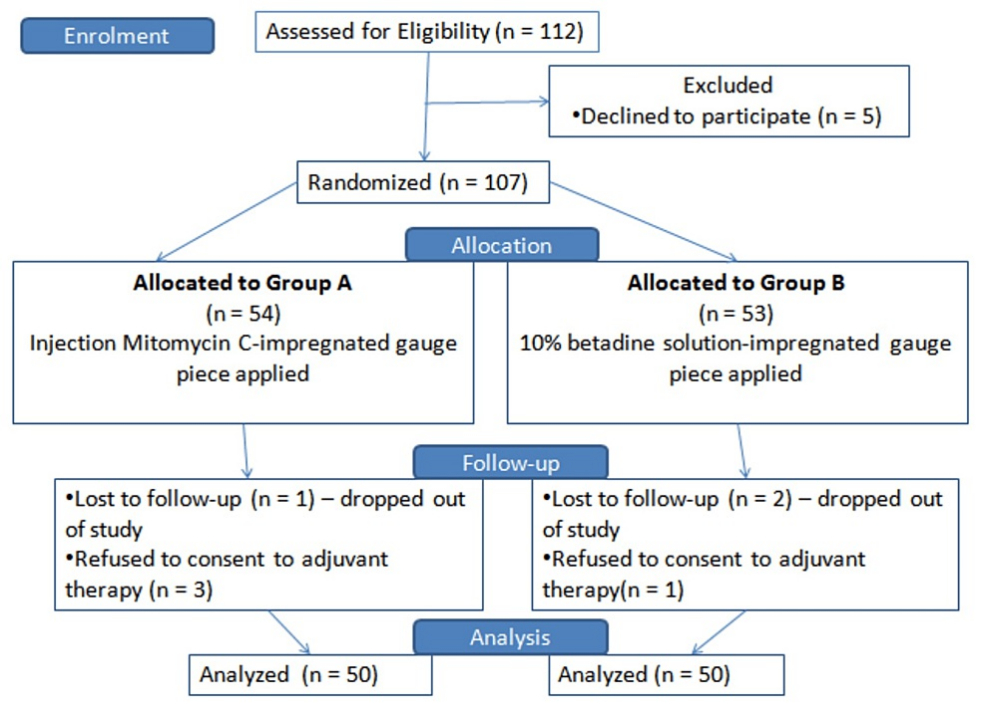
Diagram illustrating the enrolment, allocation, follow-up, and analysis of study participants.

Initial study participants included 112 individuals with oral cavity squamous cell carcinoma confirmed by biopsy. After exclusions at various phases, the final analysis (Figure 4) included 50 patients in Group A (treated with MMC) and 50 patients in Group B (treated with betadine).

In Group A (MMC group), the average age of patients was 48.02 years, while in Group B (betadine group), the average age was 49.20 years. The majority of patients in both groups were male, with 42 males and eight females in Group A and 40 males and 10 females in Group B, respectively. Thus, the age and gender distributions of the two groups were comparable (Table 1).

**Table 1:** Patient characteristics, postoperative complications, surgical resection margins, and pathological ENE in both study groups. SCC = squamous cell carcinoma; GBS = gingivobuccal sulcus; AJCC = American Joint Committee on Cancer; SSI = surgical site infection; PMMC = pectoralis major myocutaneous; ENE = extranodal extension

	Group A (mitomycin C group)	Group B (betadine group)	P-value
Mean age	48.02 years	49.20 years	
Male	42	40	0.7953
Female	8	10	
Preoperative biopsy			
Well-differentiated SCC	8	10	0.7953
Moderately differentiated SCC	38	35	0.6529
Poorly differentiated SCC	4	5	1.0000
Primary disease subsite			
Buccal mucosa	25	28	0.6889
Central arch	5	2	0.4360
Lower GBS	6	9	0.5766
Alveolus	8	9	1.0000
Lip	1	1	1.5051
Retromolar trigone	3	0	0.2424
Tongue	2	1	1.0000
AJCC cancer staging			
I	0	0	
II	02	01	1.0000
III	06	07	1.0000
IVA	24	30	0.3158
IVB	18	12	0.2752
Complications			
Skin flap necrosis (with SSI)	8	3	0.1997
PMMC flap necrosis	2	2	1.3827
Surgical resection margins			
>5 mm	44	46	0.7407
2–5 mm	6	4	0.7407
Involved	0	0	
Pathological ENE	18	12	0.2752

Regarding the oral cavity sub-site of the primary disease, there were no statistically significant differences between the groups (Table 1). The majority of patients in both groups were diagnosed with buccal mucosa cancer, followed by alveolar and lower gingivobuccal sulcus cancers.

The majority of patients in both groups had locally advanced T4 disease, while a few had N2 lymph node status (Stage IVA). In addition, a small percentage of patients (Table 1) displayed Stage IVB disease with N3b (extensive lymph node involvement). In total, 18 (36%) patients in Group A and 12 (24%) patients in Group B had N3b nodal status, signifying Stage IVB disease.

Our study explored minor as well as major complications associated with the local application of MMC. Minor complications, specifically blackening of the skin flap in the neck resulting in surgical site infections (SSIs), were observed in 16% (eight patients) of the MMC group and in 6% (three patients) of the betadine group patients. However, there was no statistically significant difference between the two groups (p = 0.1997). Two of the eight patients in the MMC group required split-thickness skin grafting (STSG) for wound closure, while the other six required debridement and secondary suturing. In contrast, primary wound debridement and closure were performed on all three patients in the betadine group without the need for STSG.

The incidence of major complications, such as PMMC flap failure or dehiscence leading to the formation of an oro-cutaneous fistula, was the same in the MMC and betadine groups at 4% (p = 1.3827). In addition to intravenous antibiotics for infection control, these cases were treated with serial debridement and regular dressings of the oro-cutaneous fistula. As soon as the infection subsided, the patients underwent revision flap surgery under general anesthesia, i.e., reconstruction of the forehead flap.

Of note, none of the patients experienced neutropenia or other systemic adverse effects as a result of MMC administration.

Locoregional recurrences

Patients underwent follow-up for a period of 15 months during the course of our study. In the MMC group, two (4%) patients experienced locoregional recurrences at three months, four (8%) patients at six months, six (12%) patients at nine months, eight (16%) patients at 12 months, and 10 (20%) patients at 15 months of follow-up. In contrast, locoregional recurrences occurred in two (4%) patients in the betadine group at three months, six (12%) patients at six months, nine (18%) patients at nine months, 12 (24%) patients at 12 months, and 15 (30%) patients at 15 months (Table 2).

**Table 2:** The incidence of locoregional recurrences in both groups during the follow-up period.

Follow-up duration	Group A (mitomycin C group), total number of patients (%)	Group B (betadine group), total number of patients (%)	P-value
3 months	2 (4%)	2 (4%)	1.0000
6 months	4 (8%)	6 (12%)	0.7407
9 months	6 (12%)	9 (18%)	0.5766
12 months	8 (16%)	12 (24%)	0.4539
15 months	10 (20%)	15 (30%)	0.3558

**Figure 5:**
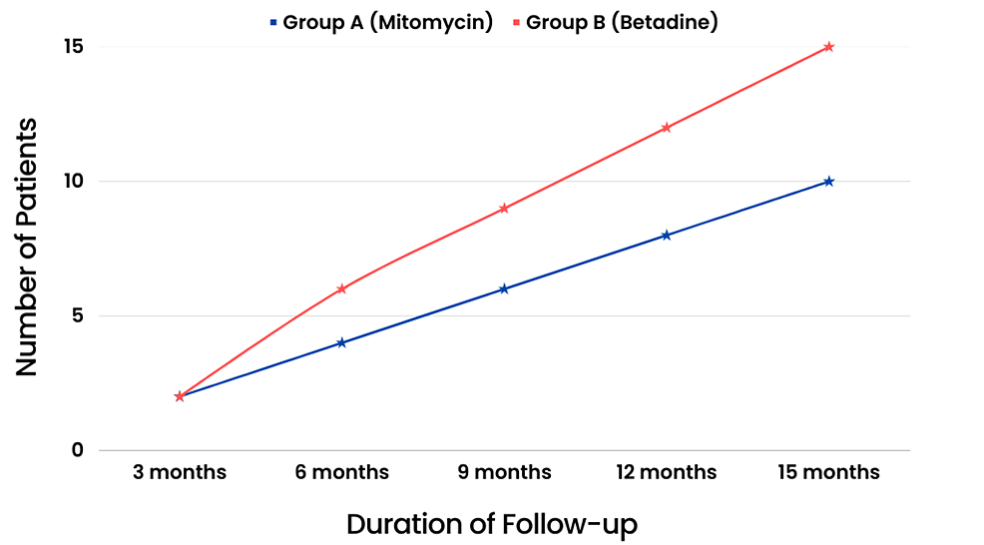
Locoregional recurrence trends in both groups during the follow-up period.

Although the difference in locoregional recurrences between the two groups was not statistically significant, there was a trend of decreasing locoregional recurrences in the MMC group relative to the betadine group as the duration of follow-up increased (Figure 5).

Among the 25 patients with locoregional recurrences, the majority, consisting of 22 (88%) patients, exhibited pathological extranodal extension (ENE). In Group A (MMC group), all 10 patients with locoregional recurrence had N3b (Stage IVB) disease with ENE, while in Group B (betadine group), 12 patients had N3b (Stage IVB) disease with ENE, two patients had N2 (Stage IVA) disease, and one patient had T4aN1 (Stage IVA) disease. In the subgroup analysis of patients with pathological ENE, only 10 of 18 patients with ENE in Group A (55.55%) experienced a recurrence, whereas all 12 patients with ENE in Group B (100%) experienced a recurrence within the same time frame. This difference in locoregional recurrence rates between the two groups was statistically significant, with a p-value of 0.0100.

## Discussion

Oral cancer, which is the sixth most prevalent malignancy worldwide [6], exhibits variable incidence rates across various geographical regions, with countries such as India, France, and South/Southeast Asia having particularly high rates [7]. Particularly prevalent in India, where it accounts for one-third of all cases worldwide, oral cancer presents a significant public health challenge as the nation undergoes economic transition. Oral cancer in India affects a sizeable proportion of the population, which is a cause for serious concern. Exposure to exogenous carcinogens, such as smokeless tobacco and alcohol consumption, is one of the primary risk factors for oral cancer.

In our study involving 100 patients with locally advanced oral cancer, we found that over 50% of the cases had buccal mucosa as the primary subsite of cancer, which is consistent with the findings of a previous study by Malik et al. [8] on oral cancer trends. All patients had a history of consuming smokeless tobacco, and some also had a history of alcoholism.

In this study, patient age ranged from 31 to 74 years, and similar to a study by Asthana et al. [9], the majority of oral cancer patients in both categories (80% of cases) were male. This finding is consistent with the observed gender difference in oral cancer cases, with males exhibiting a higher incidence of tobacco-related cancer.

In the two groups, the majority of patients had locally advanced tumors (T4/N2/N3 disease). In fact, 84% of patients in both groups were classified as stage IV. There were no statistically significant differences between the two groups in terms of age distribution, primary disease subsite, or disease stage.

MMC is a *Streptomyces*-derived antitumor antibiotic. When metabolically activated through enzymatic reduction, it produces a substance that can bind to and cross-link DNA, which contributes to its ability to inhibit tumor growth [5]. MMC is commonly administered intravesically, or directly into the bladder, to prevent the recurrence of bladder tumors in patients undergoing transurethral resection of bladder tumors (TURBT). After TURBT, MMC is infused into the bladder to target any remaining cancer cells and reduce the risk of bladder tumor recurrence. In addition, it has been extensively researched for hyperthermic intraperitoneal chemotherapy, for which MMC is the most widely used drug. Intraperitoneal MMC therapy is a well-established method for treating specific malignancies, such as peritoneal carcinomatosis and pseudomyxoma peritonei. By directly administering MMC into the peritoneal cavity, it can target cancer cells present on the peritoneal surface or in the peritoneal fluid, exerting a local cytotoxic effect. This therapeutic modality has shown efficacy in appendiceal, colorectal, and gastric cancers, frequently in conjunction with other medications [10]. Given the effectiveness of MMC in intraperitoneal therapy and its potential to prevent locoregional recurrences, it was deemed appropriate to examine its use as a local treatment on surgical resection beds in patients undergoing oral cancer surgery. This study aimed to assess the efficacy of MMC in reducing locoregional recurrences in this patient population.

In oral cancer surgeries, the prevalence of microscopic residual disease in the surgical site remains a concern despite the excision of the primary tumor. It is possible for small clusters or single cancer cells to go undetected despite meticulous surgical techniques. These residual cancer cells can serve as a potential source of locoregional recurrence, posing a significant management challenge for patients with a high nodal burden [11]. To address this issue, our study investigated the potential benefits of MMC applied directly to the surgical bed. It has been demonstrated that MMC has potent cytotoxic properties against cancer cells, inhibiting their proliferation and promoting cell death. It is hypothesized that by administering MMC locally, residual cancer cells within the surgical bed can be effectively targeted and eradicated, thereby decreasing the risk of locoregional recurrence.

Recurrence rates of oral cancer range from 18% to 76% in patients who receive conventional treatment, and it is considered the leading cause of low survival rates. The majority of studies have confirmed that the median time to recurrence after treatment is 7.5 months, and 86% of recurrences occur within 24 months [12]. Recurrences are classified as local, regional (recurrences within the lymph nodes of the neck), or distant (recurrences outside the cervical region). Local and regional recurrences are responsible for up to 90% of post-surgery and radiotherapy treatment failures [12,13].

Patients with recurrent carcinomas present a challenging clinical scenario that makes determining the most effective treatment options extremely difficult. Only a small subset of patients are candidates for salvage surgery, which is associated with dismal survival rates ranging from 30% to 45% [13]. For patients who are ineligible for salvage surgery or re-irradiation, chemotherapy remains the most effective treatment option. Even though drug combinations are improving, the prognosis for these patients remains dismal because postoperative and postradiotherapy fibrosis makes it impossible to get the therapeutic doses of the medications to the recurrent tumor [14]. In light of these obstacles, our study aimed to identify a novel therapeutic adjunct for the prevention of locoregional recurrence. As a potential therapeutic strategy, we investigated the local application of MMC injection on the surgical resection bed. Targeting the residual tumor cells within the surgical site and preventing locoregional recurrences were the motivations behind this strategy.

The presence of metastatic cervical lymph nodes is the most significant adverse prognostic factor in patients with oral cancer. ENE is a notably reliable predictor of disease recurrence and mortality [4]. A pathological ENE is the extension of metastatic carcinoma from a lymph node through its fibrous capsule and into the surrounding connective tissue. In clinical staging, ENE is determined through a physical examination and imaging techniques, which assess factors such as skin infiltration, muscle invasion, dense tethering to nearby structures, or nerve dysfunction. A positive result indicates the presence of ENE [15]. Analysis of survival in the study cohort by Tirelli et al. [16] confirmed its impact on survival. In fact, the three-year overall survival rate for this ENE-positive group was 43.2%, which was lower than that documented in the medical literature. In fact, ENE itself is a criterion for disease progression, independent of the size and location of the primary tumor [16]. In addition, ENE is a significant determinant of the intensification of adjuvant therapy (the addition of chemotherapy to radiation therapy). All of our patients with histopathologically confirmed pathological ENE received concurrent adjuvant chemoradiation in accordance with NCCN recommendations.

During the course of our study, a comprehensive 15-month follow-up was conducted for the patients. Locoregional recurrences occurred in two (4%) patients in the MMC group at three months, four (8%) patients at six months, six (12%) patients at nine months, eight (16%) patients at 12 months, and 10 (20%) patients at the 15-month mark. Similarly, two (4%) patients in the betadine group experienced locoregional recurrences at three months, six (12%) patients at six months, nine (18%) patients at nine months, 12 (24%) patients at 12 months, and 15 (30%) patients at the conclusion of the 15-month follow-up period. A notable trend was observed, although the difference in the incidence of locoregional recurrences between the MMC and betadine groups did not attain statistical significance. The MMC group consistently had fewer locoregional recurrences than the betadine group as the duration of follow-up increased. This trend suggests that MMC may have the potential to reduce the risk of locoregional recurrences over time. Nonetheless, additional research with larger sample sizes is required to confirm these findings and establish statistical significance.

Within the scope of our investigation, we discovered a strong association between locoregional recurrences and the presence of ENE, specifically N3b disease. Among the total of 25 patients who experienced locoregional recurrences, the majority, comprising 22 (88%) patients, exhibited ENE. The remaining three patients also demonstrated a high nodal burden, either in the form of N2 disease (two patients) or T4aN1 disease (one patient). This highlights the significance of identifying and addressing this particular characteristic of oral cancer in patients.

The subgroup analysis of patients with ENE showed that administering MMC locally to the surgical resection bed had several benefits. In Group A (MMC group), the proportion of patients with ENE who experienced locoregional recurrence within 15 months was considerably lower than in Group B (betadine group). In particular, only 10 out of 18 (55.56%) patients in Group A with ENE experienced recurrence, whereas all 12 (100%) patients in Group B with ENE experienced recurrence during the same time period. With a p-value of 0.0100, this difference in locoregional recurrence rates between the two groups was statistically significant. These results indicate that directly applying MMC to the surgical resection site, as done in Group A, greatly decreased local recurrences in patients with a high lymph node burden, particularly those with ENE. These results suggest the potential benefits of incorporating MMC as a therapeutic strategy to improve outcomes in this patient subgroup.

Minor complications, specifically blackening of the skin flap of the neck leading to SSI, occurred in 16% (eight patients) of the MMC group and in 6% (three patients) of the betadine group. Two patients in the MMC group required STSG for wound closure, while six patients required only debridement and secondary suturing. All three patients in the betadine group had their neck wounds debrided and closed without the need for STSG. Even though the MMC group had a higher incidence of minor complications such as blackening of the skin flap and SSI in the neck, the difference was not statistically significant (p = 0.1997). These minor complications may be effectively treated with conservative methods, such as resuturing or the use of STSG for closure, when necessary. The manageability of these complications suggests that, despite their slightly higher incidence in the MMC group, they can be effectively treated without major adverse consequences.

There was no difference in the occurrence of major complications between the two groups. Two patients in each group, i.e., the MMC group and the betadine group, developed an oro-cutaneous fistula as a result of PMMC flap failure. These major complications were managed through a series of interventions, including serial debridement and regular dressings of the oro-cutaneous fistula, as well as the administration of intravenous antibiotics to control infection. Subsequently, the patients underwent revision flap surgery under general anesthesia, specifically forehead flap reconstruction. The similarity in the occurrence of major complications emphasizes that both treatment approaches were associated with comparable risks in relation to these specific complications.

Even after a comprehensive search of the English-language medical literature, no studies on the use of chemotherapeutic agents locally on surgical resection beds to prevent locoregional recurrences of oral cancer were found. In the available literature, there are no published studies or clinical trials addressing this specific approach.

It is essential to acknowledge the limitations inherent in this study, as they can impact the interpretation of the findings. First, the sample size, especially after exclusions, was relatively small, potentially limiting the generalizability of the findings. Moreover, the follow-up period of 15 months was relatively short, which could restrict the assessment of long-term outcomes. In addition, the single-center design of the study may introduce biases and limit the generalizability of the findings to a larger population. To address these limitations and obtain more robust evidence, future research should consider multicenter studies with larger sample sizes, longer follow-up periods, and the exploration of diverse treatment options. Such studies would yield a more comprehensive understanding of the subject and enhance the generalizability of the findings.

## Conclusions

Our study demonstrated that the local administration of MMC on surgical resection beds may lower the risk of locoregional recurrences in patients with oral cancer, especially those with ENE. These findings contribute to ongoing efforts to enhance treatment strategies and patient outcomes for this challenging malignancy. Despite being the first study of its kind, our research contributes to the existing body of knowledge on the management of oral cancer by investigating the use of chemotherapeutic agents on surgical resection beds. Future multicenter studies with larger sample sizes, extended follow-up periods, and a variety of treatment options should be conducted to provide more robust evidence and broaden the applicability of our findings.
